# Examination of the effect of spinal anesthesia informational video on anxiety level and postoperative analgesia in transurethral resection of prostate

**DOI:** 10.3389/fmed.2025.1660177

**Published:** 2025-10-14

**Authors:** Bilal Atilla Bezen, Kamil Taşkapılı, Veli Mert Yazar, Osman Gerçek, Berkay Eren

**Affiliations:** ^1^Department of Anesthesiology, Faculty of Medicine, Afyonkarahisar Health Science University, Afyonkarahisar, Türkiye; ^2^Department of Urology, Faculty of Medicine, Afyonkarahisar Health Science University, Afyonkarahisar, Türkiye

**Keywords:** preoperative anxiety, spinal anesthesia, informational video, TURP, intraoperative sedation, postoperative analgesia

## Abstract

**Objectives:**

To evaluate the effect of an informational video on spinal anesthesia in reducing preoperative anxiety and postoperative analgesic requirements in patients undergoing TURP surgery.

**Materials and methods:**

This clinical trial was conducted on 93 urologic patients, who underwent transurethral resection of prostate with spinal anesthesia. The patients were randomly divided into two groups using a website: Group 1 who received informational video about spinal anesthesia at least 2 h before the operation in addition to standard verbal and written information, Group 2 who received standard verbal and written information. The baseline anxiety levels of all patients were evaluated in the preoperative anesthesia outpatient clinic (T0). A second time, anxiety levels were measured in the preoperative preparation room (T1). Intraoperative sedation levels were monitored with an OAASS score of 3–4. The amount of the additional dexmedetomidine administered was recorded. Postoperative 0th, 2nd, 4th, 6th, 12th, and 24th hour VAS scores and the rescue analgesic time and amount were recorded.

**Results:**

No significant difference was found between the groups in terms of STAI-T, STAI-S0, APAIS-Anxiety0, APAIS-Information0 measured in the preoperative outpatient clinic evaluation (*p* > 0.05). STAI-S1, APAIS-Anxiety1, and APAIS-Information1 results assessed in the preoperative preparation room were found to be significantly lower in Group 1 (*p* = 0.001, for each). When the median amount of additional dexmedetomidine per kilogram was compared, 0.10 μg/kg (IQR: 0.09) were used in Group-1, while 0.30 μg/kg (IQR: 0.17) were used in Group-2. Intraoperative additional dexmedetomidine requirement was significantly lower in Group 1 (*p* = 0.001). Although the amount and timing of rescue analgesic use did not differ between the groups, the proportion of patients requiring rescue analgesia within 24 h was significantly lower in Group 1 (*p* = 0.284, *p* = 0.926, *p* = 0.033, respectively).

**Conclusion:**

In the preoperative period, the spinal anesthesia information video reduces preoperative anxiety more than standard verbal and written information. Additionally, the spinal anesthesia information video also reduces the amount of sedative agents used intraoperatively.

## Introduction

Preoperative anxiety is a prevalent emotional response, occurring in up to 80% of cases, characterized by discomfort and tension that may adversely affect the perioperative phase (1). Preoperative anxiety can adversely impact patient experience, physiological responses to the operation, and postoperative recovery (1–3). It is attributed to factors such as fears of not awakening and experiencing pain before and after surgery. Moreover, a lack of adequate information about the anesthesia and surgical procedure contributes to increased anxiety (4).

In urological procedures such as transurethral resection of prostate (TURP), which are typically performed on older male patients, preoperative anxiety is often heightened by age-related comorbidities and concerns about anesthesia (5). Although spinal anesthesia is often preferred over general anesthesia for its advantages, including effective postoperative analgesia, it may still raise concerns among patients due to misconceptions and insufficient information (6). Using educational videos before anesthesia correlates with enhanced patient satisfaction, greater knowledge retention, and less perioperative anxiety across diverse surgery groups (7, 8).

Audiovisual resources that clearly explain the anesthetic procedure—including its steps, expected sensations, and potential side effects—can enhance patient understanding of spinal anesthesia and help alleviate common apprehensions. Although interest in patient-centered education is growing, evidence regarding its impact on the context of TURP surgery remains limited.

This study aimed to evaluate the effect of an informational video on spinal anesthesia in reducing preoperative anxiety and postoperative analgesic requirements in patients undergoing TURP surgery. Audiovisual education is hypothesized to enhance perioperative outcomes by reducing anxiety and improving the effectiveness of analgesia. The findings are intended to support the integration of patient-centered educational tools into routine anesthetic practice.

## Methods

This prospective, randomized clinical trial was conducted in Afyonkarahisar Health Science University Hospital’s anesthesiology reanimation, and urology clinics. All human-related procedures complied with the ethical principles outlined in the 2013 Declaration of Helsinki and were approved by the relevant institutional ethics committee.

Following the approval of the Afyonkarahisar Health Science University Clinical Research Ethics Committee (Date:01.09.2023, Number:381) male patients aged between 18 and 85 years, with an ASA physical status classification of I to III, adequate cognitive function, no diagnosed neurological or psychiatric disorders, no contraindications to spinal anesthesia, and the ability to understand Turkish, who were scheduled for elective surgery due to benign prostatic hyperplasia, were included in the study. Patients were excluded from the study if they did not meet the inclusion criteria, had visual or hearing impairments, had conditions contraindicating spinal anesthesia, or required conversion to general anesthesia during the operation. After giving their written informed consent, participants were enrolled in the study.

Demographic data (like age, weight, height, and comorbidities) of patients were recorded. The education levels of the patients were recorded in 4 groups as 1-college and above, 2-high school and above, 3-secondary school and above, 4-primary school and below. The baseline anxiety levels of all patients were evaluated in the preoperative anesthesia outpatient clinic with the Amsterdam Preoperative Anxiety and Information Score Scale (APAIS) and State Anxiety Inventory Trait (STAI-T) and State (STAI-S) questionnaires. The APAIS questionnaire assesses anxiety using four questions and evaluates the need for information with two additional items. The STAI consists of two distinct components, each comprising 20 questions: STAI-T measures a patient’s general anxiety levels, while STAI-S assesses situational anxiety (9, 10). This assessment point was designated as T0, with the corresponding scores recorded as STAI-S0 and APAIS0.

On the day of surgery, a preoperative anesthesia consultation was conducted at least 2 h before the operation. Patients were randomized into two groups. Randomization was performed by automatically assigning numbers between 1 and 98 to two groups using a web-based system^1^. Assignment results were concealed in sequentially numbered, sealed, opaque envelopes. Randomized group assignment was determined by opening the envelope by a researcher on the day of surgery, prior to debriefing. With a single-blind study design, all postoperative follow-up data were collected and evaluated by an anesthesiologist blinded to patient allocation. Patients in Group 1 were informed with a video explaining the spinal anesthesia method in addition to the standard verbal and written information form, while patients in Group 2 were informed with only the standard verbal and written information form. The primary objective of the video was to provide detailed information about the anesthesia process. A four-and-a-half-minute informational video was prepared using footage recorded with the consent of a non-participating patient before the initiation of the study. The video depicted the patient’s journey from the preoperative preparation room to the operating room, including the administration of spinal anesthesia. Also, it used animations to explain the basic anatomy of neuraxial anesthesia. The video featured explanations and demonstrations of various aspects, including the insertion of the intravenous cannula, patient positioning for neuraxial anesthesia, insertion of the neuraxial anesthetic, and the use of patient monitoring. The advantages and disadvantages of spinal anesthesia are also discussed in the video. Additionally, before starting the study, positive expert review and patient feedback were received about the informational video.

The time during which all patients were in the preoperative preparation room was designated as T1. During this period, the anxiety scores (APAIS Anxiety1, APAIS Information1, STAI-S1) were measured again, and then the patients were taken to the operating room. Then routine anesthesia monitoring was performed and spinal anesthesia was applied in the sitting position, by determining the lumbar 3–4 interval, with a 25-gauge Quincke spinal needle and 12.5 mg 0.5% hyperbaric bupivacaine. Surgery commenced after confirming the adequacy of sensory and motor blockade.

For intraoperative sedation, dexmedetomidine was administered at a dose of 0.3 μ/kg via slow intravenous infusion over 10 min, and the sedation level was systematically evaluated every 3 min using the Observer’s Assessment of Alertness Sedation Scale (OAASS). The OAASS score was adjusted to be within the range of 3–4, and if the score exceeds 4, intravenous 0.05 μ/kg of dexmedetomidine would be provided. The amount of the additional sedative drug administered was recorded.

The time patients arrived in the postoperative recovery room was designated as hour zero (0 h), and the first Visual Analog Scale (VAS) score assessment (VAS 0) was conducted at this point. Postoperative 2nd, 4th, 6th, 12th, and 24th hour VAS scores were recorded in the urology clinic. According to the standard analgesia protocol, patients received 1 gram of paracetamol in 100 mL solution twice daily. In cases where the VAS score exceeded 4 during follow-up, rescue analgesia with tramadol at a dose of 1 mg/kg was planned. The patients’ rescue analgesic requirement, the hour they first needed rescue analgesic, and the dose were recorded. Patients’ willingness to undergo spinal anesthesia again was assessed using a 5-stage Willingness Scale (1 – I would not want it at all, 5 – I would very much like it). The length of hospital stay is recorded.

Primary outcome was the difference in preoperative anxiety scores. Secondary outcomes were intraoperative additional dexmedetomidine requirement, postoperative VAS scores and rescue analgesic consumption.

### Statistical analysis

The IBM SPSS program was used in statistical analysis. Descriptive statistics were given as numbers and percentages, arithmetic mean, standard deviation, median (inter quartile range) values. Quantitative data were analyzed using the Student’s *t*-test or Mann–Whitney *U* test. Categorical data were evaluated using the chi-square test.

Since APAIS information, APAIS anxiety, and STAI-S scores were found to be normally distributed, these parameters were compared between groups using a paired samples t-test. The homogeneity of variances was assessed using the Levene test. APAIS information, APAIS anxiety, and STAI S scores over time and the effect of the video on these were examined using repeated measures analysis of variance. The Greenhouse–Geisser correction was used when the sphericity assumption was not met. Results were considered statistically significant when *p* < 0.05.

In the sample size calculation, based on a previously reported study, effect size of 0.63, a two-tailed analysis with an alpha error of 0.05 and a power of 81% indicated that 84 participants (42 per group) would be required (11). Accounting for a potential dropout rate of 15%, the study was planned to include 49 participants in each group.

## Results

**Figure 1:**
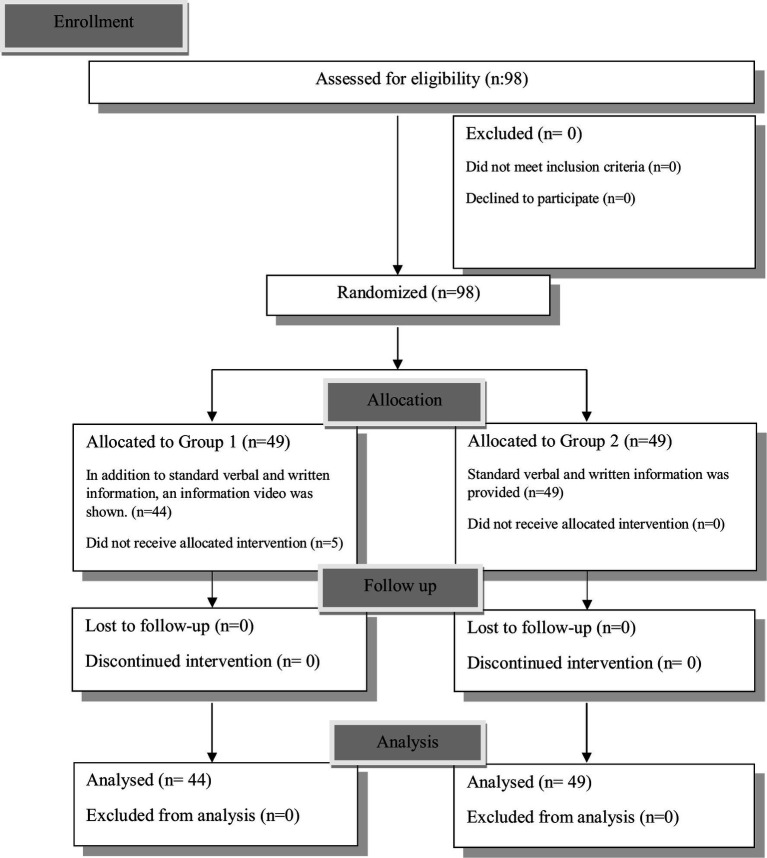
CONSORT flow chart describing participant progression through the study.

A total of 93 patients were included in the study after the exclusion of five patients in Group 1 who declined to participate in the video-based informational intervention, resulting in 44 patients (47.3%) in Group 1 and 49 patients (52.7%) in Group 2. Figure 1 shows the Consort Diagram. Demographic data of the patients were similar between the groups (*p* > 0.05) (Table 1). There was no difference between the groups in terms of data such as ASA, comorbidity, history of surgery and anesthesia, and educational status (*p* > 0.05) (Table 1).

**Table 1:** Demographic and medical characteristics of patients.

Parameters		Group-1 *n* = 44	Group-2 *n* = 49	*p*
Age (year)		66.84 ± 5.74	67.65 ± 5.82	0.501*
Weight (kg)		78.48 ± 11.61	82.18 ± 9.13	0.089*
Height (cm)		170.25 ± 5.87	172.53 ± 6.12	0.071*
ASA physical status	1	3(%6.8)	4(%7.5)	0.578**
	2	22(%50)	29(%59.2)	
	3	19(%43.2)	16(%32.7)	
Comorbidity	Yes	34(%77.3)	42(%81.7)	0.293**
	No	10(%22.7)	7(%18.3)	
Education	1	6(%13.6)	12(%24.5)	0.340**
	2	15(%34.1)	20(%40.8)	
	3	8(%18.2)	6(%12.2)	
	4	15(%34.1)	11(%22.4)	
History of surgery	Yes	33(%75)	36(%73.5)	0.866**
	No	11(%25)	13(%26.5)	
History of spinal anesthesia	Yes	18(%40.9)	15(%30.6)	0.300**
	No	26(%59.1)	34(%69.4)	

*Independent samples *T*-test, **Pearson Chi square test.

Table 2 shows comparison of anxiety scores between groups before exclusion, and STAI-T, STAI-S0, APAIS-Anxiety0, APAIS-Information0 were similar (*p* > 0.05, for each) (Table 2). After the exclusion no significant difference was found between the groups in terms of STAI-T, STAI-S0, APAIS-Anxiety0, APAIS-Information0 measured in the preoperative outpatient clinic evaluation (*p* > 0.05) (Table 3). STAI-S1, APAIS-Anxiety1, and APAIS-Information1 results assessed in the preoperative preparation room were found to be significantly lower in Group 1 (*p* = 0.001) (Table 3).

**Table 2:** Comparison of anxiety scores according to groups before exclusion.

Scores	Group-1 (*n* = 49)	Group-2 (*n* = 49)	*p*
STAI-T	43.65 ± 5.28	42.94 ± 5.40	0.510
STAI-S-0	44.69 ± 4.23	46.20 ± 4.58	0.094
APAIS-Anxiety-0	9.76 ± 3.09	9.90 ± 3.74	0.837
APAIS-Information-0	6.08 ± 1.55	6.51 ± 1.68	0.193

Independent samples *T*-test.

**Table 3:** Comparison of anxiety scores according to groups after exclusion.

Scores	Group-1 (*n* = 44)	Group-2 (*n* = 49)	*p*
STAI-T	43.41 ± 5.38	42.94 ± 5.40	0.675
STAI-S-0	44.66 ± 4.29	46.20 ± 4.58	0.098
APAIS-Anxiety-0	9.73 ± 3.23	9.90 ± 3.74	0.816
APAIS-Information-0	6.00 ± 1.59	6.51 ± 1.68	0.139
STAI-S-1	40.95 ± 4.06	44.22 ± 4.61	**0.001**
APAIS-Anxiety-1	7.30 ± 2.71	9.82 ± 3.05	**0.001**
APAIS-Information-1	3.80 ± 1.06	5.49 ± 1.54	**0.001**

Independent samples *T*-test. Bold text shows statically significance.

**Figure 2:**
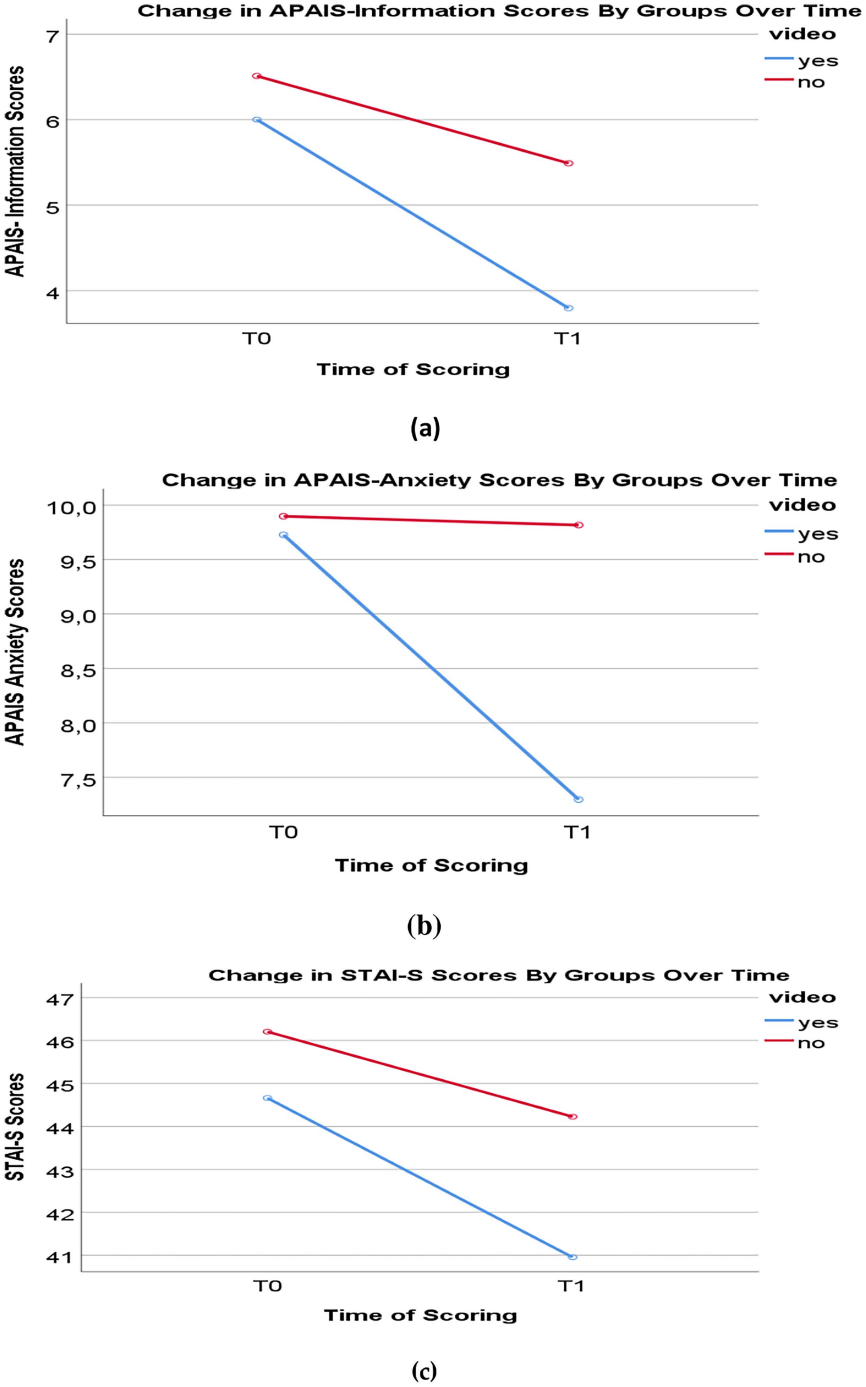
Comparison of anxiety scores by groups over time: **(a)** change in APAIS-Information Scores; **(b)** change in APAIS-Anxiety Scores; **(c)** change in STAI-S Scores.

Significant decreases were observed in APAIS-anxiety, APAIS-information, and STAI-S scores in both groups after the information session. When the effect of the groups on score changes was examined, it was observed that the score change in Group 1 was statistically significantly higher than in Group 2 (*p* < 0.05) (Table 4 and Figure 2). Interaction between groups was detected in all three measurements, with changes in scores over time.

**Table 4:** Comparison of APAIS-Anxiety, APAIS-Information, and STAI-State scores at baseline (T0) and post-intervention (T1) within and between groups.

Score	Group 1 Mean ± SD	Group 2 Mean ± SD	Within-group difference (T1–T0), *t* (p)	Between-group difference, *p*
APAIS-Anxiety	T0: 9.73 ± 3.23 T1: 7.30 ± 2.71	T0: 9.90 ± 3.74 T1: 9.82 ± 3.05	Group 1: *t* = 6.71, ***p* < 0.001** Group 2: *t* = 0.18, *p* = 0.861	T0: *p* = 0.861 T1: ***p* = 0.027**
APAIS-Information	T0: 6.00 ± 1.59 T1: 3.80 ± 1.06	T0: 6.51 ± 1.68 T1: 5.49 ± 1.54	Group 1: *t* = 9.35, ***p* < 0.001** Group 2: *t* = 5.57, ***p* < 0.001**	T0: ***p* = 0.001** T1: ***p* = 0.001**
STAI-State	T0: 44.66 ± 4.29 T1: 40.95 ± 4.06	T0: 46.20 ± 4.58 T1: 44.22 ± 4.61	Group 1: *t* = 5.41, ***p* < 0.001** Group 2: *t* = 5.50, ***p* < 0.001**	T0: ***p* = 0.024** T1: ***p* = 0.005**

Paired samples *T*-test. Bold text shows statically significance.

When the groups were compared in terms of duration of operation and the number of patients who received additional dexmedetomidine, the results were found to be similar (*p* = 0.452, *p* = 0.868, respectively). The amount of additional dexmedetomidine per kilogram was significantly lower in Group 1 (median: 0.10 μg/kg, IQR: 0.09) compared with Group 2 (median: 0.30 μg/kg, IQR: 0.17). Mann–Whitney *U* test indicated a highly significant difference (*U* = 65.5, *Z* = –5.03, *p* < 0.001) (Table 5). The effect size was large (r = 0.69), confirming that this finding is not only statistically significant but also clinically meaningful.

**Table 5:** Comparison of operative and postoperative data according to groups.

Parameters		Group-1 *n* = 44	Group-2 *n* = 49	*p*
Duration of operation(min)		88.07 ± 22.31	91.76 ± 24.54	0.452*
Additional sedation requirement	Yes	25(%56.8)	27(%55.9)	0.868^#^
	No	19(%43.2)	22(%44.1)	
Amount of additional sedative (μg/kg) Median; IQR		0.10;0.09	0.30;0.17	0.001**
Rescue analgesic requirement	Yes	20(%45.5)	33 (%67.3)	**0.033** ^**#**^
	No	24(%54.5)	16(%32.7)	
Amount of rescue tramadol (mg)		80.00 ± 12.24	83.18 ± 9.08	0.284*
First rescue analgesic time (h)		8(2–24)	8(1–24)	0.926**
Length of hospital stay (day)		1.86 ± 0.70	2.10 ± 0.73	0.116*
Willingness score		3.55 ± 1.15	3.47 ± 1.08	0.744*

*Independent *T*-test, ^#^Chi-Square Test, **Mann–Whitney *U* test. Bold text shows statically significance.

Additionally, the number of patients requiring rescue analgesic within the first 24 h postoperatively was significantly lower in Group 1 (*p* = 0.033). Among patients who required postoperative rescue analgesia, the mean tramadol consumption was 80.0 ± 12.2 mg in Group 1 and 83.2 ± 9.1 mg in Group 2. The difference between groups was not statistically significant [*t*(51) = −1.08, *p* = 0.284; mean difference −3.18, 95% CI –9.09 to 2.72]. The effect size was small (Cohen’s *d* ≈ 0.29), and no clinically meaningful difference was observed. And the first rescue analgesic usage time was similar (*p* = 0.926) (Table 5).

Although the length of hospital stay was shorter in Group 1, it was not statistically significant (*p* = 0.116). When compared in terms of willingness score, no significant difference was found between the groups (*p* = 0.744) (Table 5).

**Figure 3:**
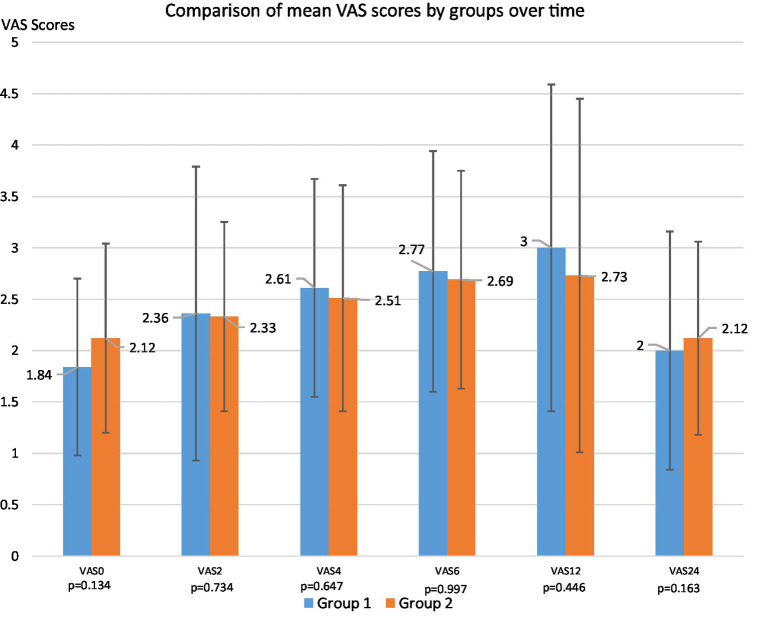
Comparison of mean VAS scores by groups over time (VAS, Visual Analog Scale).

No significant differences in VAS scores were observed between the groups at any time point (*p* > 0.05) (Figure 3).

## Discussion

In our study, preoperative anxiety scores decreased in both groups after the information about spinal anesthesia. However, the primary outcome of our study is that the spinal anesthesia information video significantly reduced preoperative anxiety scores compared to standard written and verbal information. Although spinal anesthesia is sufficient for TURP surgery, sedative agents are used together to enhance patient comfort. Additionally, another significant finding of our study, which also evaluated the use of intraoperative sedative agents, is that the spinal anesthesia informational video reduced the need for dexmedetomidine during surgery. The approximately 60% reduction in dexmedetomidine dose in the video group may contribute to enhanced patient safety and faster recovery. Previous studies mainly compared preoperative anxiety scores with postoperative anxiety scores, which limits their ability to demonstrate the effects on preoperative anxiety. The methodology of our study differs in this regard.

Factors such as gender, type of surgery, patient’s surgical history, ASA classification, and literacy affect preoperative anxiety (12). In a study conducted by Batuman et al. (13) in pediatric patients, it was observed that preoperative information videos reduced preoperative anxiety and postoperative negative behaviors. Li et al. (14) found that individualized preoperative information reduced perioperative anxiety more than standard information. In a study by Sagir et al. (15), patients exposed to visuals explaining spinal anesthesia, similar to our study, showed reduced anxiety scores. Similarly, in our study, video-based information reduced anxiety scores more than standard written and verbal information and decreased the number of patients using postoperative analgesics.

In a study on preoperative anxiety in TURP surgeries, midazolam and information were compared, and it was found that preoperative information was as effective as midazolam (5). However, the use of anxiolytics like midazolam in elderly patients has decreased because it increases the incidence of postoperative delirium (3). In our study, the amount of dexmedetomidine per kilogram used for intraoperative sedation was found to be lower in the video-watching group.

There are studies showing that preoperative anxiety scores are positively correlated with postoperative pain scores (16, 17). High anxiety scores lead to an increase in the doses of anesthetic and analgesic agents used during the perioperative period (18). In the study by Bayrak et al. (19), patients with high anxiety scores had an increased need for postoperative analgesics. In our study, the group that watched the spinal anesthesia informational video had lower anxiety scores, and the number of patients using postoperative rescue analgesic decreased.

In our study, patients who watched the video had lower anxiety scores; however, their postoperative VAS scores were not significantly different from those of the standard information group. Notably, the proportion of patients requiring postoperative rescue analgesia was lower in the video group. This finding may be explained by the higher rate of rescue analgesic use observed in the group that received standard verbal and written information.

This study was conducted in a single center with a Turkish-speaking male patient population undergoing a specific urologic procedure. Therefore, the generalizability of our results to other populations, female patients, or different surgical contexts may be limited. Another limitation of our study is the relatively short postoperative follow-up period. While this was sufficient to evaluate early analgesic efficacy, it does not allow conclusions regarding longer-term outcomes such as sustained patient satisfaction, chronic pain development, or delayed adverse effects. The other limitations of this study include a relatively small sample size, the subjective nature of the anxiety assessment scales, and the use of an informational video that has not been validated in previous studies. Future multicenter studies with more diverse patient groups and extended follow-up periods are warranted to validate our findings and provide a more comprehensive evaluation of their applicability and longer-term outcomes. Future research should also include qualitative assessments to better capture the patients’ perceptions and emotional responses.

## Conclusion

In the preoperative period, the spinal anesthesia information video reduces preoperative anxiety more than standard verbal and written information. Additionally, the spinal anesthesia information video also reduces the amount of sedative agents used intraoperatively.

## Data Availability

The original contributions presented in the study are included in the article/supplementary material, further inquiries can be directed to the corresponding author.

## Footnotes

^1^ www.randomizer.org
